# Dyslipidemia and Diabetes Increase the OPG/TRAIL Ratio in the Cardiovascular System

**DOI:** 10.1155/2016/6529728

**Published:** 2016-12-14

**Authors:** Barbara Toffoli, Bruno Fabris, Giacomo Bartelloni, Fleur Bossi, Stella Bernardi

**Affiliations:** Department of Medical Sciences, University of Trieste, Cattinara Teaching Hospital, Strada di Fiume 447, 34149 Trieste, Italy

## Abstract

*Background*. Dyslipidemia and diabetes are two of the most well established risk factors for the development of cardiovascular disease (CVD). Both of them usually activate a complex range of pathogenic pathways leading to organ damage. Here we hypothesized that dyslipidemia and diabetes could affect osteoprotegerin (OPG) and TNF-related apoptosis-inducing ligand (TRAIL) expression in the vessels and the heart.* Materials and Methods*. Gene and protein expression of OPG, TRAIL, and OPG/TRAIL ratio were quantified in the aorta and the hearts of control mice, dyslipidemic mice, and diabetic mice.* Results*. Diabetes significantly increased OPG and the OPG/TRAIL ratio expression in the aorta, while dyslipidemia was the major determinant of the changes observed in the heart, where it significantly increased OPG and reduced TRAIL expression, thus increasing cardiac OPG/TRAIL ratio.* Conclusions*. This work shows that both dyslipidemia and diabetes affect OPG/TRAIL ratio in the cardiovascular system. This could contribute to the changes in circulating OPG/TRAIL which are observed in patients with diabetes and CVD. Most importantly, these changes could mediate/contribute to atherosclerosis development and cardiac remodeling.

## 1. Introduction

In industrialized countries, cardiovascular disease (CVD) remains the predominant cause of morbidity and mortality. It is current scientific opinion that CVD occurs as a part of a series of events that form a continuum [1]. The first stage of this continuum corresponds to the presence of risks factors that predispose to tissue injury, such as hypertension, dyslipidemia, and diabetes [2]. These risk factors usually activate a complex range of pathogenic pathways leading to the development of atherosclerosis and ischemic heart disease. Failure to effectively manage any stage of this continuum results in ventricular hypertrophy, heart failure, end-stage heart disease, and cardiovascular death.

One of the molecular pathways that could be activated in response to these risk factors and be involved in CVD development is that of osteoprotegerin (OPG) and TNF-related apoptosis-inducing ligand (TRAIL), as reviewed in [3]. OPG and TRAIL are both members of the TNF superfamily, and they are widely expressed in different tissues, including the heart and the vessels, in health and disease states [4, 5]. Although initially OPG actions seemed to be limited to bone metabolism [6] and those of TRAIL to host defense against tumors [7], recent studies have suggested that both molecules might actually be involved in CVD development and progression [3].

Epidemiological studies have shown that there is an association between circulating OPG, TRAIL, and CVD. OPG was found to be directly correlated [5, 8, 9], while TRAIL was inversely correlated, with cardiovascular morbidity and mortality [10, 11]. Likewise, OPG was significantly associated with the level of C-reactive protein [12], which is an independent predictor of acute vascular events and adverse outcomes, while TRAIL was negatively correlated with it [10]. Recently, Secchiero and colleagues have shown that patients with coronary artery disease have an increased OPG/TRAIL ratio, which further increases in patients developing heart failure [13]. Consistent with this epidemiological data, experimental studies suggest that OPG and TRAIL have opposite actions, as OPG has proinflammatory [12] and proatherogenic effects [14, 15], while TRAIL seems to be anti-inflammatory [16, 17] and antiatherogenic [18–20].

Based on these premises, we hypothesized that dyslipidemia and diabetes, which are two well known risk factors for CVD, could modify the vascular and cardiac expression of OPG and TRAIL, leading to an increased OPG/TRAIL ratio at a tissue level. The aim of this study was to explore the vascular and cardiac changes of OPG and TRAIL in experimental models of dyslipidemia and diabetes.

## 2. Materials and Methods

### 2.1. Animal Model and Experimental Protocol

A total of 12 male C57BL/6J (CNT) mice and 24 male apolipoprotein E null (ApoE^−/−^) mice on a C57BL/6J background, aged 6 weeks, were studied for 14 weeks. At 6 weeks of age, ApoE^−/−^ mice were further randomized to saline (ApoE) or streptozotocin (ApoE + DM), which were delivered intraperitoneally in five consecutive daily doses. The daily dose of streptozotocin (STZ) (Sigma-Aldrich) was 55 mg/kg of body weight. After one week, blood glucose was checked and only the mice with blood glucose levels greater than 15 mM were included in the ApoE + DM group. During the study period, all the mice were fed with a standard diet. The animals were kept in a temperature-controlled room (22 ± 1°C) on a 12 h light/12 h dark cycle with free access to food and water and they were fed ad libitum for the length of the study. After 14 weeks, the animals were anesthetized with an intraperitoneal injection of thiobutabarbital sodium (Inactin, Sigma-Aldrich) (60 mg/kg body weight) and sacrificed by exsanguination via cardiac puncture. Bloods and tissues (aortas and hearts) were collected for further analysis.

This study was carried out at the Animal House of the University of Trieste. All the animal experiments were approved by the animal ethics committee of the University of Trieste (ID 28.0.2008) and the Italian Ministry of Health, conforming to the Guide for the Care and Use of Laboratory Animals.

### 2.2. General Parameters

At the end of the study, we measured body weight and systolic blood pressure (SBP). In particular, SBP was assessed by a computerized noninvasive tail cuff system in conscious mice. Glucose, total cholesterol, HDL cholesterol, and triglycerides were measured in the sera by autoanalyzer technique (Hitachi 917, Tokyo, Japan). Circulating levels of OPG and TRAIL were measured in the sera by ELISA (R&D Systems DuoSet #DY459 and #DY1121).

### 2.3. Aortic and Cardiac Structural Changes

In half of the animals in each group, aortae were collected and placed in 10% neutral-buffered formalin for atherosclerosis quantification, before being processed for immunohistochemical analyses. In the other half, aortae were snap-frozen in liquid nitrogen and stored at −80°C for RNA extraction and gene expression analyses. Plaque area was quantified as described previously [15]. Briefly, aortae were cleaned of excess fat and stained with Sudan IV-Herxheimer's solution. Then, they were dissected longitudinally and pinned flat onto wax. Images were acquired with a dissecting microscope (Olympus BX-50) equipped with a high-resolution camera (Sony XC-77CE). Digitized images were then evaluated with an image analysis system (Image-Pro Plus 6.3 software, Media Cybernetics). Total plaque area was quantified as the percentage area of aorta stained red.

As for the hearts, they were divided and half was put in formalin and half was put in liquid nitrogen. The part that was fixed in formalin was embedded in paraffin, cut in 4 *μ*m thick sections, and stained with hematoxylin and eosin (H&E) in order to evaluate cardiomyocyte hypertrophy. Cardiomyocyte hypertrophy was assessed by measuring the shortest diameter of 200 cells, which were selected when they showed the spindle-shaped transverse section including the elliptical nucleus. The shortest transverse diameter was measured 3 times per cell, and the values were averaged, as reported by [21]. The other half that was snap-frozen in liquid nitrogen was used either for gene expression analyses or for immunohistochemical stainings.

### 2.4. Quantitative Real-Time RT-PCR

Gene expression of OPG and TRAIL was determined by real-time quantitative RT-PCR (reverse transcription-polymerase chain reaction) in aortic and cardiac tissue. In order to isolate mRNA from the aorta and the heart, tissue was homogenized and processed as previously reported [22]. Then, mRNA was treated with the DNase inactivation reagent (Ambion DNA-free product #AM-1906), and 3 *μ*g of treated mRNA was subsequently used to synthesize cDNA with Superscript First Strand synthesis system for RT-PCR (Gibco BRL). The gene expression of OPG and TRAIL was analyzed by real-time quantitative RT-PCR using the TaqMan system (Life Technologies) for OPG and the SYBR Green system (Life Technologies) for TRAIL. Fluorescence for each cycle was quantitatively analyzed by an ABI Prism 7900HT Sequence Detection System (Applied Biosystems). Gene expression of OPG was normalized to 18S mRNA, while that of TRAIL was normalized to Rps9. Results were reported as ratio compared with the level of expression in untreated controls, which were given an arbitrary value of 1.

### 2.5. Immunohistochemistry

The presence of OPG and TRAIL in the aortae was evaluated by immunostainings on 4 *μ*m thick paraffin sections. In particular, after antigen retrieval in citrate buffer (pH6) endogenous peroxidase was quenched for 10 minutes using 3% H_2_O_2_ in PBS. To localize OPG, we used a biotinylated goat anti-OPG antibody (R&D Systems, 1 : 10 dilution) followed by a catalyzed signal amplification system (Dako). To localize TRAIL, we used a monoclonal mouse anti-TRAIL antibody (R&D Systems, 1 : 50 dilution), applied overnight at 4°C. Biotinylated goat anti-mouse immunoglobulins (Vector Laboratories, dilution 1 : 200) were then used as secondary antibody, followed by streptavidin-HRP (Dako).

The presence of OPG and TRAIL in the hearts was evaluated by immunostaining on 5 *μ*m thick frozen sections. After neutralization of endogenous peroxidase, sections were incubated overnight with the goat anti-OPG antibody (R&D Systems, 1 : 10 dilution) and the monoclonal mouse anti-TRAIL antibody (R&D Systems, 1 : 50 dilution). Biotinylated immunoglobulins, diluted 1 : 200, were then applied as secondary antibodies, followed by streptavidin-HRP (Dako). In both tissues, the staining was visualized by reaction with 3,3′-diaminobenzidine tetrahydrochloride (Sigma-Aldrich). After counterstaining with hematoxylin, all the sections were examined by light microscopy (Olympus BX50WI) and digitized using a high-resolution camera (Q-Imaging Fast 1394). The proportional area of brown staining was measured by an image analysis system (Image-Pro Plus 6.3 software, Media Cybernetics) in order to quantify OPG and TRAIL expression in aortae and hearts.

### 2.6. Statistical Analysis

Results are expressed as means ± standard error of the mean. Differences in the mean among groups were analyzed using two-way ANOVA. Pairwise multiple comparisons were made using Bonferroni posttest analysis. *p* < 0.05 was considered statistically significant.

## 3. Results

### 3.1. General Parameters

The general parameters of the mice are reported in Table 1. ApoE^−/−^ mice had higher levels of total cholesterol and triglycerides as compared to the CNT mice. In line with the study protocol, the mice in the ApoE + DM group were hyperglycemic. Moreover, diabetes was associated with a significant reduction of body weight and a significant increase of total cholesterol as compared to nondiabetic ApoE^−/−^ mice.

### 3.2. Structural Changes in the Aorta and the Heart

**Figure 1:**
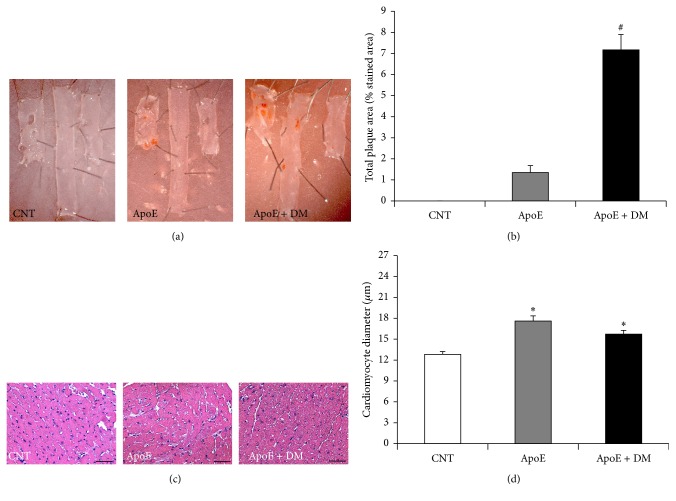
Effect of dyslipidemia and diabetes on atherosclerosis and cardiomyocyte hypertrophy. (a) Representative en face aortic sections showing atherosclerosis. (b) Percentage of the aorta stained red with Sudan IV in each group. Result from CNT is not shown, as there was no plaque present. (c) Representative H&E-stained hearts (original magnification 25x) and (d) related cardiomyocyte size quantification (in *μ*m). Scale bar = 50 *μ*m. Data show mean ± SEM. ^*∗*^ *p* < 0.05 versus CNT; ^#^ *p* < 0.05 versus ApoE. CNT is for C57BL/6J mice; ApoE is for ApoE^−/−^ mice; ApoE + DM is for ApoE^−/−^ mice + diabetes mellitus.

ApoE^−/−^ mice had a significant increase in total plaque area, which increased further after the induction of diabetes (Figures 1(a) and 1(b)). As for cardiac changes, dyslipidemia was associated with a significant increase in cardiomyocyte size (Figures 1(c) and 1(d)), indicating cardiomyocyte hypertrophy, with no further increase after the induction of diabetes.

### 3.3. OPG and TRAIL Gene Expression in the Aorta and the Heart

**Figure 2:**
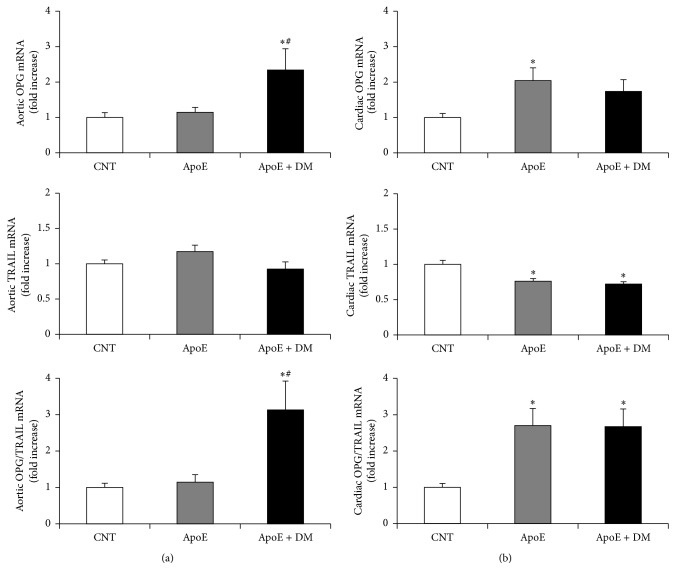
Effect of dyslipidemia and diabetes on OPG, TRAIL gene expression, and OPG/TRAIL ratio in the aorta and the heart. (a) Aortic messenger RNA expression of OPG, TRAIL, and OPG/TRAIL ratio, reported as relative gene units. (b) Cardiac messenger RNA expression of OPG, TRAIL, and OPG/TRAIL ratio, reported as relative gene units. Data are expressed as mean ± SEM. ^*∗*^ *p* < 0.05 versus CNT; ^#^ *p* < 0.05 versus ApoE. CNT is for C57BL/6J mice; ApoE is for ApoE^−/−^ mice; ApoE + DM is for ApoE^−/−^ mice + diabetes mellitus.

In the aorta, the induction of diabetes was associated with a significant increase in OPG gene expression and in the OPG/TRAIL ratio, while dyslipidemia had no effect on OPG/TRAIL ratio as compared to controls (Figure 2(a)). By contrast, in the heart, dyslipidemia was the major determinant of OPG/TRAIL tissue changes, not only by increasing OPG but also by reducing TRAIL gene expression. So dyslipidemia increased significantly cardiac OPG/TRAIL ratio, with no further differences after the induction of diabetes (Figure 2(b)).

### 3.4. OPG and TRAIL Protein Expression in the Aorta and the Heart

**Figure 3:**
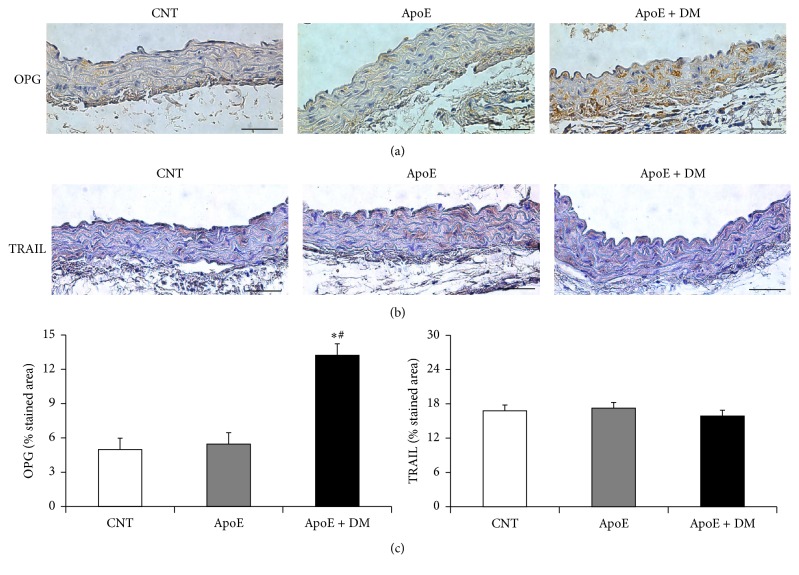
Effect of dyslipidemia and diabetes on aortic OPG and TRAIL protein expression. (a) Representative sections of aorta immunostained for OPG and (b) TRAIL (original magnification 25x). (c) Aortic OPG and TRAIL protein expression reported as percentage stained area. Scale bar = 50 *μ*m. Data show mean ± SEM. ^*∗*^ *p* < 0.05 versus CNT; ^#^ *p* < 0.05 versus ApoE. CNT is for C57BL/6J mice; ApoE is for ApoE^−/−^ mice; ApoE + DM is for ApoE^−/−^ mice + diabetes mellitus.

Immunostainings showed that diabetes significantly increased aortic OPG expression as compared to the other groups, while TRAIL was unchanged (Figure 3). On the other hand, in the heart, it was dyslipidemia that significantly increased OPG protein expression, with no further changes after the induction of diabetes. As for cardiac TRAIL, TRAIL protein expression decreased in the ApoE and ApoE + DM groups (data not shown).

### 3.5. Circulating OPG and TRAIL

The levels of circulating OPG mirrored its vascular changes. Circulating OPG was 1.91 ± 0.21 ng/mL in the control group, 2.05 ± 0.1 ng/mL in the ApoE group, and 3.1 ± 0.19 in the ApoE + DM group, such that OPG increased significantly in diabetic mice as compared to the other groups (*p* < 0.05, ApoE + DM versus CNT and versus ApoE group). Dyslipidemia alone had no effect on OPG circulating levels. As for TRAIL, we were unable to measure its circulating levels, as they were below the detection levels of the ELISA we used. It has already been argued that OPG, which circulates at much higher levels than its ligands (RANKL and TRAIL), may be a more stable overall measure of OPG/TRAIL activity than TRAIL [23].

## 4. Discussion

Epidemiological studies report associations between OPG, TRAIL, and CVD. Patients with coronary artery disease have an increased OPG/TRAIL ratio, which further increases in the group of patients who develop heart failure [13]. Dyslipidemia and diabetes are two of the most firmly established risk factors for CVD [2]. Here, we evaluated the effects of dyslipidemia, alone and in combination with diabetes, on aortic and cardiac OPG and TRAIL expression and found that while diabetes is the major determinant of OPG/TRAIL tissue changes in the vessels, dyslipidemia is it in the heart.

This data is in line with previous experimental works showing that diabetes increased OPG expression and the OPG/TRAIL ratio in the aorta [24] and that the onset of diabetes was associated with an increase of circulating OPG [25]. Consistent with these observations, in our study, we found that aortic and circulating OPG increased significantly only in the group of mice with diabetes. Taken together, these findings seem to support the hypothesis that the vessels might be the source of increased circulating levels of OPG in patients with diabetes and/or CVD.

It has to be noted that the increase of circulating and tissue OPG could be not only a risk marker but also a risk factor for atherosclerosis and CVD development. Firstly, animal models point to a role for OPG in glucose metabolism regulation, as OPG injections increased significantly glucose levels [12, 26], which represent one of the risk factors for CVD. This effect could be mediated by TRAIL blockade, as TRAIL lowers glucose levels [16]. By offsetting TRAIL effects, OPG could also promote body weight gain and dyslipidemia [20], as well as atherosclerotic plaque development [18, 20]. Secondly, several studies have shown that OPG has proinflammatory and profibrotic effects on the vasculature. Consistent with these actions, our group has shown that OPG increases leukocyte adhesion to endothelial cells [14], that it increases TGF-beta-mediated fibrogenesis and proliferation in vascular smooth muscle cells [27], and that it increases atherosclerosis extension in diabetic ApoE^−/−^ mice [15].

By contrast, dyslipidemia alone did not affect OPG/TRAIL ratio in the vasculature. This data is consistent with a few in vitro studies showing that oxidized LDL did not change OPG expression in human coronary artery smooth muscle cells [28] and lymphocytes [29]. Nevertheless, in our study, we found that dyslipidemia significantly increased OPG/TRAIL ratio in the heart, while diabetes had no additional effect on it.

It has been shown that dyslipidemia is independently associated with left ventricle (LV) hypertrophy [5, 30, 31]. This was confirmed by experimental studies on ApoE^−/−^ mice, where dyslipidemia was associated with increased LV mass in the absence of hemodynamic stress [32]. Consistent with previous reports, here we found that dyslipidemia was associated with cardiomyocyte hypertrophy. This data suggests that hyperlipidemia might be an early initiator of cardiac remodeling. It has been demonstrated that triacylglycerol overload and the increased availability of other lipid intermediates, such as ceramides, diacylglycerol, and oxidized phospholipids, can all activate inflammatory pathways and oxidative stress, leading to cellular apoptosis and myocardial scarring [33, 34].

It is possible that the increase in cardiac OPG/TRAIL ratio that was associated with dyslipidemia represents one of the mediators of lipid-induced cardiac remodeling. As for OPG, a recent study has demonstrated that OPG delivery induced LV hypertrophy, while, in vitro, cardiomyocytes transfected with AAV-OPG increased dramatically the expression of hypertrophy-related proteins, such as ANP, *α*-MHC, and troponin I [35]. In the cardiac fibroblast, OPG overexpression led to a significant increase in the fibrosis-related proteins [35]. By contrast, we showed that TRAIL administration to mice with diabetic cardiomyopathy had cardioprotective effects, as it reduced cardiac fibrosis and apoptosis, which are generally contributing to cardiac remodeling [36].

In conclusion, here we found that both dyslipidemia and diabetes affect OPG/TRAIL ratio in the cardiovascular system. This could contribute to the changes in circulating OPG/TRAIL which are observed in patients with diabetes and CVD. Most importantly, these changes could mediate/contribute to atherosclerosis development and cardiac remodeling.

## Figures and Tables

**Table 1:** General parameters.

Parameter	CNT	ApoE	ApoE + DM
Body weight (g)	30.89 ± 0.74	33.46 ± 1.40	24.86 ± 0.61^*∗*#^
LV/BW (mg/g)	3.42 ± 0.09	3.57 ± 0.06	3.29 ± 0.07^#^
SBP (mmHg)	81.57 ± 8.31	86.11 ± 5.12	87.33 ± 3.78
Fasting glucose (mmol/L)	9.48 ± 1.17	11.94 ± 0.91	54.77 ± 11.73^*∗*#^
Total cholesterol (mmol/L)	2.60 ± 0.35	11.97 ± 0.92^*∗*^	21.10 ± 3.94^*∗*#^
Triglycerides (mmol/L)	0.84 ± 0.19	1.28 ± 0.14^*∗*^	1.70 ± 0.37^*∗*^
Cholesterol HDL (mmol/L)	1.91 ± 0.24	1.86 ± 0.15	2.60 ± 0.51

^*∗*^ *p* < 0.05 versus CNT; ^#^ *p* < 0.05 versus ApoE; LV, left ventricle; BW, body weight.
